# Prolonged zinc exposure modulates biofilm metabolic activity and conjugation in *Enterococcus faecalis*

**DOI:** 10.1186/s12866-026-05250-x

**Published:** 2026-06-04

**Authors:** Denisa Fenclova, Kristyna Hrazdilova, Martina Coufalova, Josy ter Beek, Ronnie Per-Arne Berntsson, Ludek Zurek, Kristyna Cihalova

**Affiliations:** 1 Department of Chemistry and Biochemistry, Faculty of AgriSciences, Mendel University in Brno, Brno, Czechia; 2 Department of Medical Biochemistry and Biophysics, Umeå University, Umeå, SE-90187 Sweden; 3 Wallenberg Centre for Molecular Medicine & Umeå Centre for Microbial Research, Umeå University, Umeå, Sweden; 4 Department of Microbiology, Nutrition and Dietetics, Czech University of Life Sciences, Prague, Czechia

**Keywords:** *Enterococcus faecalis*, Zinc oxide, Nanoparticles, Conjugation, Horizontal gene transfer, Biofilm, Aggregation, Long-term adaptation

## Abstract

**Background:**

Zinc oxide (ZnO), including its nanoparticulate form (ZnONPs), is widely used in agriculture and accumulates in the environment, where it may impose sustained selective pressure on microbial communities. However, the impact of prolonged zinc exposure on horizontal gene transfer and conjugation dynamics in *Enterococcus faecalis* remains poorly understood.

**Results:**

We exposed *Enterococcus faecalis* OG1RF:pCF10 (donor) and OG1SSp (recipient) to prolonged zinc exposure (20 serial passages) and analyzed phenotypic and transcriptional changes associated with conjugation and virulence-related traits. Chronic exposure to ZnO and ZnONPs was associated with pronounced aggregation in the plasmid-carrying donor strain, reduced optical density values, and significantly lower recoverable CFU/mL at 24 h, although extensive clumping likely affected CFU recovery. Zinc exposure was also associated with increased metabolic activity within established biofilms, while gelatinase production and antibiotic susceptibility remained unchanged. ZnONP-adapted recipient cells showed a significant increase in conjugation frequency, whereas ZnO-adapted recipients and zinc-adapted donors showed non-significant upward trends. Notably, transcription of genes within the plasmid-encoded *prgQ* conjugation operon was increased even in the absence of exogenous pheromone stimulation. In contrast, short-term zinc exposure did not enhance plasmid transfer, indicating that increased conjugation required long-term adaptation rather than acute stress.

**Conclusions:**

These findings indicate that prolonged zinc exposure is associated with altered aggregation, biofilm-associated metabolic activity, and conjugation dynamics in *E. faecalis*. However, the underlying mechanisms remain unresolved and may involve a combination of physiological, regulatory, and genetic adaptations arising from long-term exposure.

**Supplementary Information:**

The online version contains supplementary material available at 10.1186/s12866-026-05250-x.

## Background

Horizontal gene transfer (HGT) is a major driver of bacterial adaptation, facilitating dissemination of antibiotic resistance genes, virulence factors, and metabolic traits across microbial communities [1–3]. Conjugation is one of the most efficient HGT mechanisms in both Gram-negative and Gram-positive pathogens, including *Enterococcus faecium*, *Klebsiella pneumoniae*, and *Pseudomonas aeruginosa* [4, 5]. In enterococci, conjugative plasmids and other mobile genetic elements contribute to the dissemination of antimicrobial resistance and virulence-associated traits [6–8]. This is clinically relevant in *Enterococcus faecalis*, an opportunistic and nosocomial pathogen whose ability to horizontally acquire and disseminate resistance determinants, including genes coding for vancomycin resistance, represents a serious clinical threat [7–9]. While antibiotics are established inducers of conjugation [10], environmental stressors, particularly heavy metals, may likewise enhance plasmid transfer and contribute to antimicrobial resistance (AMR) dissemination in agricultural and clinical environments [11–13].

The pheromone-responsive plasmid pCF10 provides a well-characterized model system to study conjugation dynamics in *E. faecalis* [14–16]. pCF10 encodes a type IV secretion system (T4SS) responsible for DNA transfer [17]. Its expression is regulated by the PQ promoter, which is controlled by the balance between the inducing pheromone cCF10, encoded by chromosomal *ccfA*, and the inhibitory pheromone iCF10, encoded by *prgQ*, the first gene of the *prgQ* operon on pCF10 [18–20]. Both pheromones signal through the regulator PrgX and maintain tight control of conjugation [14–16]. Basal transcription of *prgQ* produces two shorter transcripts, QS and QL, that prevent premature activation of the conjugation machinery, whereas readthrough transcription generates the full transcript required for expression of downstream transfer genes [21]. Additional regulators, including PrgY and PrgU, further limit self-induction and prevent toxic overexpression of the aggregation substance PrgB. The pCF10-encoded aggregation substance PrgB plays a central role in cell aggregation, biofilm formation, mating, and virulence [22]. Therefore, factors that interfere with pheromone signaling, aggregation, biofilm-associated growth, and/or donor–recipient interactions may influence plasmid transfer in this species.

Heavy metals persist in soils and impose sustained selective pressure on microbial populations [23]. At sub-inhibitory concentrations, they can promote adaptive responses including biofilm formation, virulence, and horizontal gene exchange [11, 12, 24]. Within this context, zinc represents potentially important environmental stressor. Zinc contamination is strongly associated with swine production, where zinc oxide (ZnO) has been used at pharmacological doses (2,000–3,000 mg/kg feed) to control post-weaning diarrhea [25]. Although the European Union reduced permitted levels to 150 mg/kg in 2022 [26], high-dose ZnO remains widely applied elsewhere [27], leading to zinc accumulation in agricultural soils [28, 29]. Such environments may serve as reservoirs of adapted strains capable of entering clinical settings via food chains or water systems [30, 31]. Similar eco-clinical links have been observed for other heavy metals, where adaptations acquired outside hospitals enhanced pathogen survival in healthcare settings [32, 33].

Zinc is a redox-inert transition metal essential for bacterial physiology, where it contributes to enzymatic activity, gene regulation, and oxidative stress responses [34, 35]. At elevated concentrations, however, zinc disrupts membrane integrity, destabilizes proteins, and perturbs cellular homeostasis [36, 37]. Both ZnO and ZnONPs have been reported to alter bacterial physiology and gene expression in diverse Gram-negative and Gram-positive species. Reported effects include increased membrane permeability, leakage of intracellular components, and strain-dependent changes in biofilm development ranging from enhanced matrix production to inhibition [6, 38, 39].

Particle size influences zinc bioavailability and biological effects [40]. As an alternative to bulk ZnO, zinc oxide nanoparticles (ZnONPs) are increasingly used in agriculture and antimicrobial applications due to their high surface reactivity [41, 42]. Due to their smaller particle size and increased surface reactivity, ZnONPs may exert biological effects distinct from ZnO [40–42]. Associations between metal stress and enhanced conjugation have been reported mainly in Gram-negative bacteria [43, 44], and long-term zinc exposure was shown to induce persistent phenotypic and molecular adaptations in *Escherichia coli* [45], including increased virulence and multidrug resistance.

Although pCF10 regulation is well defined, the impact of chronic zinc exposure on pheromone-controlled conjugation remains unclear. Transcriptomic studies in *E. faecalis* have demonstrated zinc-responsive changes in transport systems and mobile genetic elements [18], suggesting potential crosstalk between metal stress responses and pheromone-regulated transfer. However, whether prolonged zinc exposure can modulate the pheromone-responsive pCF10 conjugation system and thereby influence horizontal gene transfer dynamics has not been experimentally demonstrated.

**Fig. 1:**
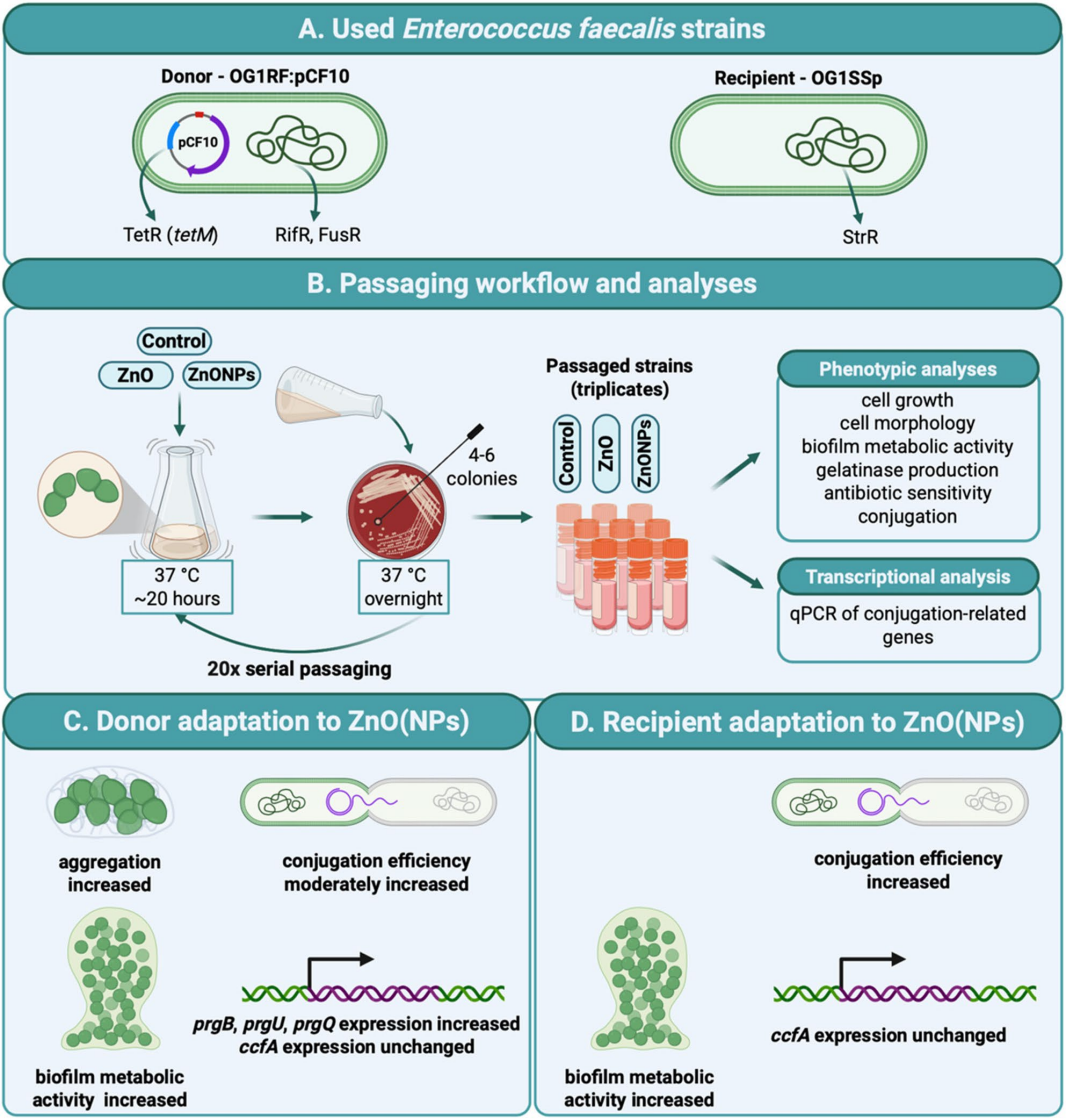
Experimental design and overview of phenotypic and transcriptional responses of *Enterococcus faecalis* to long-term zinc exposure. (A) *Enterococcus faecalis* strains used in this study: the donor strain OG1RF:pCF10 carrying the pheromone-responsive plasmid pCF10 (with tetracycline resistance) and with chromosomal resistance to rifampicin and fusidic acid, and the plasmid-free recipient strain OG1SSp resistant to streptomycin. (B) Experimental workflow. Donor and recipient strains were subjected to 20 serial passages in the presence of ZnO or ZnONPs, or under control conditions without zinc exposure. Passaging was performed in three independent biological replicates, generating three independently evolved lineages per condition. After adaptation, strains were cultivated in the absence of zinc and analyzed for phenotypic traits and gene expression. (C) Responses of zinc-adapted donor cells (OG1RF:pCF10). Long-term zinc exposure was associated with increased cell aggregation, increased metabolic activity within established biofilms, and increased transcription of conjugation-related genes within the *prgQ* operon (*prgB* and *prgU*), with a moderate increase in *prgQ* transcription and a trend toward increased conjugation efficiency. (D) Responses of zinc-adapted recipient cells (OG1SSp). Zinc exposure increased conjugation efficiency and metabolic activity within established biofilms, while transcription of the pheromone gene *ccfA* remained unchanged. Created in BioRender: https://BioRender.com/f8goadc

Here, we investigated whether long-term exposure to ZnO or ZnONPs alters conjugation dynamics in *E. faecalis*. Using donor strain OG1RF:pCF10 and recipient strain OG1SSp, we examined phenotypic traits, conjugation efficiency, and transcriptional responses of key genes within the *prgQ* operon following prolonged zinc exposure. An overview of the experimental design and main analyses performed in this study is shown in Fig. 1.

## Materials and methods

### Serial passaging of bacteria with zinc

*Enterococcus faecalis* strain OG1RF:pCF10 was kindly provided by Dr. Ronnie P.-A. Berntsson at the Umeå University, Sweden, and *E. faecalis* OG1SSp was kindly provided by Dr. Lynn Hancock at the University of Kansas, USA. *Enterococcus faecalis* OG1RF:pCF10 is chromosomally resistant to rifampicin and fusidic acid and carries the pCF10 plasmid with the *tetM* gene (tetracycline resistance), whereas OG1SSp is a plasmid-free recipient strain with chromosomal resistance to streptomycin. These strains were used to investigate zinc-induced horizontal gene transfer. Four to six well-isolated colonies grown on Columbia blood agar (CBA; Lab Media Servis, Czech Republic) at 37 °C, were resuspended in phosphate-buffered saline (PBS; Thermo Fisher Scientific, Waltham, MA, USA) and adjusted to OD₆₀₀ nm = 0.15 AU. *Enterococcus faecalis* OG1RF:pCF10 was cultured in Brain Heart Infusion (BHI; Oxoid, UK) broth with tetracycline (8 mg/L), and *E. faecalis* OG1SSp in BHI with streptomycin (4 mg/mL). After each streaking step, 4–6 well-isolated colonies were randomly selected and pooled to inoculate the subsequent liquid culture to preserve population heterogeneity and reduce the likelihood of selecting single random mutants.

Zinc oxide (ZnO; Oxoid, Netherlands) and zinc oxide nanoparticles (ZnONPs; Oxoid, USA) were suspended in 0.2% (v/v) dimethyl sulfoxide (DMSO; Merck, Germany) in HPLC-grade water, sonicated (5 min), and added to cultures to a final concentration of 2 mg/mL. Controls received 0.2% DMSO only.

Cultures were incubated at 37 °C for ~ 20 h with shaking, streaked onto CBA and incubated overnight. This cycle was repeated 20× to obtain ZnO-, ZnONPs-, and DMSO- (control-) passaged strains. All passaging was performed in biological triplicates, generating three independently evolved lineages per condition. These lineages were maintained separately and used as independent biological replicates in all subsequent assays. The passaged strains were used for all assays described in this study, which were performed in the absence of ZnO or ZnONPs. The only exception was the short zinc exposure conjugation experiment (see details below).

### Scanning electron microscopy (SEM)

**Fig. 2:**
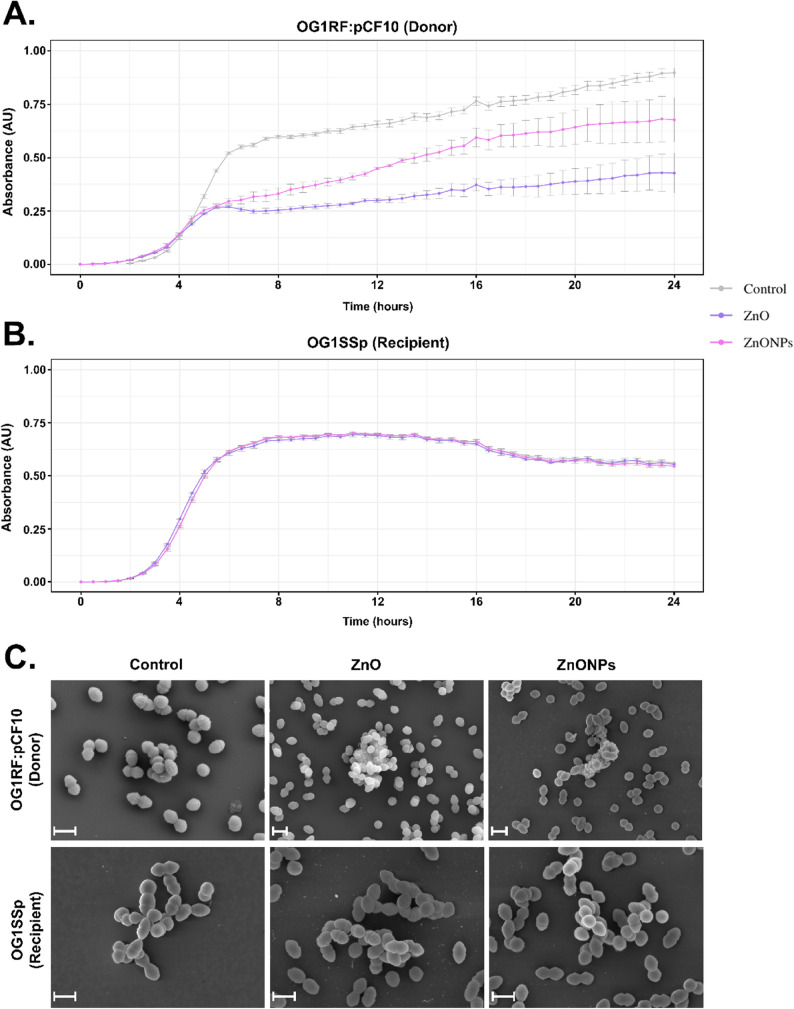
Optical density and morphology of *E. faecalis* after long-term zinc exposure. **A**, **B** Bacterial growth measured by optical density (OD₆₀₀) of OG1RF:pCF10 and OG1SSp over 24 h in Mueller–Hinton broth after 20 serial passages. Growth was monitored at 37 °C using a Bioscreen C analyzer, and measurements were performed in the absence of ZnO or ZnONPs. Data are presented as mean ± SEM of three independently evolved lineages. **C** Scanning electron microscopy of OG1RF:pCF10 and OG1SSp strains grown in normal BHI medium, after 20 passages without ZnO (control), with ZnO or with ZnONPs. Representative fields were selected to visualize extracellular matrix-like structures; therefore, there are differences in magnification between panels, white scale bars represent 1 μm

For SEM analysis, one representative strain from each of the three independently evolved lineages was selected for each condition (untreated, ZnO-treated, ZnONPs-treated) for both donor and recipient strains. 20 mL BHI with either 10 mg/L tetracycline (for donor strains) or 1 mg/mL streptomycin (for recipient strains) were inoculated 1:50 from an overnight culture, one for each of the passaged conditions, and incubated for 3.5 h at 37 °C and 180 rpm. The 6 cultures were spun down at 3,000 × g, 4 °C for 10 min. Pellets were resuspended in 1 mL 0.1 M Na-phosphate buffer (pH 7.4) and transferred to a 1.5 mL tube. The cells were again pelleted, but now at 20,000 × g, 4 °C, 10 min. All supernatant was removed, and the pellets were carefully resuspended in 600 µL 0.1 M Na-phosphate buffer (pH 7.4). Afterward, 600 µL 5% glutaraldehyde in 0.1 M Na-phosphate buffer (pH 7.4) was added, mixed by pipetting up and down to crosslink the cells, and incubated for 1 h at 4 °C. 600 µL of the mixtures were then further incubated overnight at 4 °C on top of polylysin-coated coverslips that were placed at the bottom of a 24-well plate. The coverslips were washed, dried, coated and imaged as described in Sun et al. [46]. Cell width was quantified from the SEM images shown in Fig. 2C using Fiji [47]. Each image was calibrated individually using the image-specific pixel size. For each condition, 20 cells with clearly visible boundaries were measured across the widest visible part of the cell, perpendicular to the longest cell axis when cells appeared ovoid. Cells for which the width could not be reliably determined because of extensive overlap, partial visibility, or coverage by extracellular material were excluded. Statistical comparisons between each zinc-exposed group and the untreated control were performed using an unpaired t-test in GraphPad Prism v.8.

### Bacterial growth analysis

*Enterococcus faecalis* OG1RF:pCF10 and OG1SSp were cultivated overnight on CBA at 37 °C. Colonies were resuspended in Mueller-Hinton (MH) broth (Oxoid, Hampshire, UK) and adjusted to OD₆₀₀ nm of 0.15 AU. Cultures were then diluted 1:100 in fresh MH broth, and 100 µL (~ 1 × 10⁶ CFU/mL) were transferred into Honeycomb 2 microplate. Growth was monitored at 37 °C for 24 h using a Bioscreen C analyzer (Oy Growth Curves Ab Ltd., Helsinki, Finland), with OD₆₀₀ readings every 30 min with shaking. Plates were shaken for 10 s prior to each measurement (maximum amplitude, normal speed), followed by a 5 s pause before absorbance reading. For CFU-based growth measurements, overnight cultures grown on CBA at 37 °C were adjusted to an OD₆₀₀ of 0.15 AU and diluted 1:100 in fresh MH broth. The experiment proceeded for 24 h, and samples were collected at 0, 5, 10, 15, and 24 h. Serial dilutions were prepared and plated on BHI agar containing rifampicin and tetracycline using the drop-plate method, with three 10-µL technical replicate drops per dilution. Plates were incubated at 37 °C, and colonies were counted after 24 h.

### Biofilm formation

The biofilm assay was adapted from protocol for the 7-day microtiter plate-based method described by Rihacek et al. [45], with modifications in cultivation medium, starting inoculum, and replicate structure. Overnight cultures were diluted in BHI broth with 1% glucose to OD₆₀₀ = 0.15 AU. 200 µL of each culture were inoculated into 96-well U-bottom polystyrene plates (Falcon, Corning, USA) in eight technical replicates and incubated statically at 37 °C for 7 days to allow for mature biofilm formation. Medium was replaced daily. Eight uninoculated wells containing the same sterile medium were included as negative controls to monitor for potential contamination. After incubation, the wells were washed 3× with PBS, and metabolic activity within established biofilms was assessed using Alamar Blue. The working solution was prepared by diluting the reagent 1:9 (v/v) in PBS, and 200 µL were added per well (final concentration 10% v/v). Fluorescence was measured at an excitation wavelength of 560 nm and an emission wavelength of 590 nm. Each lineage was tested in eight technical replicates.

### Gelatinase activity

Overnight cultures on CBA were resuspended in PBS to OD₆₀₀ = 0.15 AU. One microliter of suspension was spotted onto Slanetz-Bartley (S-B) agar (Lab Media Servis, Czech Republic) with 1.5% skimmed milk. Plates were incubated statically at 37 °C for 24 h. Gelatinase activity was evaluated by the presence and diameter of clear halos around colonies. The presence of the *gelE* gene was confirmed by PCR amplification using previously described gene-specific primers [48].

### Antibiotic susceptibility

Antibiotic susceptibility was tested by disk diffusion on MH agar. Overnight cultures on CBA were resuspended in PBS to OD₆₀₀ = 0.15 AU. 100 µL of suspension were spread evenly on MH agar plates. Antibiotic disks (Oxoid, UK) were applied using an automated disk dispenser (Oxoid Antimicrobial Susceptibility Test System ST6090). Tested antibiotics, selected according to EUCAST guidelines [49] for their clinical relevance against *E. faecalis*, included: penicillin G (6 µg), ampicillin (10 µg), erythromycin (15 µg), clindamycin (2 µg), tetracycline (30 µg), trimethoprim/sulfamethoxazole (25 µg), gentamicin (10 µg), vancomycin (30 µg), and teicoplanin (30 µg). Plates were incubated at 37 °C for 24 h (aerobic). Inhibition zones were measured with a digital caliper. Each lineage was plated separately.

### Conjugation assay with passaged strains

Conjugation was performed between OG1RF:pCF10 (rifampicin- and tetracycline-resistant donor) and OG1SSp (streptomycin-resistant recipient). Experiments were conducted in two setups: (i) treated donor (ZnO, ZnONPs, or control) + untreated recipient, and (ii) treated recipient + untreated donor. Overnight cultures on BHI agar with antibiotics were adjusted to OD₆₀₀ = 0.15 AU, diluted 1:100 in BHI, incubated 2.5 h at 37 °C/150 rpm. Donor and recipient were mixed 1:1 and incubated another 2.5 h. Serial dilutions were prepared and plated on BHI agar containing streptomycin and tetracycline using the drop-plate method, with three 10-µL drops applied per dilution. Plates were incubated at 37 °C, and colonies were counted after 24 h for assays with treated recipients, or after up to 48 h for assays with treated donors, to ensure visible and countable transconjugant colonies. Conjugation frequency = transconjugants/donor cells (T/D).

### Short-term zinc exposure conjugation assay

To investigate the effect of short-term zinc exposure, conjugation was also carried out between the non-passaged OG1RF:pCF10 (donor; fusidic acid and tetracycline resistant) and OG1SSp (recipient; streptomycin resistant). Overnight cultures on BHI agar with antibiotics were resuspended in PBS to OD₆₀₀ = 0.15 AU, diluted 1:100 in BHI, incubated 2.5 h at 37 °C/105 rpm with either ZnO, ZnONPs (both at 2 mg/mL in 0.2% DMSO), or with a 0.2% DMSO control. Donor and recipient cells with the same additions (either ZnO, ZnONP or control) were mixed 1:1 and incubated for another 2.5 h. Serial dilutions were plated on BHI agar with: donor selection (fusidic acid and tetracycline), recipient (streptomycin), transconjugant (streptomycin and tetracycline). Colonies were counted after 24–48 h at 37 °C. Conjugation frequency = T/D.

### RNA isolation, cDNA synthesis, and quantitative real-time PCR (qPCR)

Overnight cultures were grown on CBA, resuspended in BHI to OD₆₀₀ = 0.15 AU, diluted 1:100 and incubated for approximately 5 h at 37 °C with shaking (180 rpm) until mid-exponential phase. For induced control conditions, the donor strain OG1RF:pCF10 and the recipient strain OG1SSp from the untreated passaged control lines (maintained without zinc exposure) were cultured in BHI supplemented (1:1, v/v) with cell-free supernatant from the respective untreated counterpart strain. Supernatants were obtained from 5 h cultures grown to mid-exponential phase, centrifuged, and sterile-filtered (0.22 μm) prior to use. Supernatants were mixed 1:1 with fresh BHI to minimize potential nutrient depletion effects. Untreated passaged strains grown in fresh BHI without supernatant supplementation served as controls. These control cultures were not exposed to zinc. Cells were harvested (1,500 × g, 15 min, 4 °C), washed with PBS, and processed for RNA extraction using TRIzol (Invitrogen). The lysis protocol included an additional 10-min incubation at − 20 °C immediately after homogenization. DNA was removed with TURBO DNA-free™ (Ambion). RNA concentration and purity were determined using a NanoDrop OneC spectrophotometer (Thermo Fisher Scientific). Purified RNA samples were stored at − 80 °C. First-strand cDNA was synthesized from 500 ng of DNase-treated RNA using the Transcriptor First Strand cDNA Synthesis Kit (Roche) with random hexamer primers in a final reaction volume of 20 µL.

Quantitative PCR was carried out on a qTOWER³ system (Analytik Jena) using LUNA^®^ Universal qPCR Master Mix (New England Biolabs) primers listed in Table 1, designed in Geneious Prime and validated using the IDT Real-Time PCR Tool. The cycling conditions were as follows: initial denaturation at 95 °C for 3 min, followed by 35 cycles of denaturation at 95 °C for 15 s and combined annealing/elongation at 60 °C for 30 s. In donor strains, expression of *ccfA*, *prgB*, *prgQ*, *prgX*, and *prgU* was analyzed, while in the recipient strain only *ccfA* expression was assessed. Each sample was analyzed in technical duplicates from three independently evolved lineages. Ct values were determined in the qTOWER software using default baseline and threshold settings and exported for downstream analysis in Microsoft Excel. PCR amplification efficiencies were calculated from raw fluorescence data using LinRegPCR [50]. The calculated efficiencies for each primer pair are reported in Table 1. Gene expression was normalized to the reference genes *recA* and *gdhA*, with weighting based on inverse variance to account for the higher stability of *gdhA* under the experimental conditions. Stability of the reference genes was evaluated based on Ct variability across all experimental conditions. As *gdhA* exhibited lower variance than *recA*, inverse variance weighting was applied during normalization. ΔCt values were calculated as Ct(target) − Ct(reference), where Ct(reference) was obtained by inverse-variance weighting of *recA* and *gdhA*. Expression levels in ZnO-treated, ZnONPs-treated, and induced control samples were compared with the untreated donor controls. Statistical significance was evaluated using unpaired t-tests on ΔCt values, and results are presented as log₂-transformed fold changes relative to the untreated control.

**Table 1:** Primer sequences, amplicon lengths, and PCR amplification efficiencies (E ± SD) for reference and target genes used in qPCR analysis. F, forward primer; R, reverse primer

Gene	Primer Sequences	Amplicon length (bp)	References	PCR efficiency (E ± SD)
*gdhA*	F: GGAATTGATGTGGCGTTAG	152	[51]	1.98 ± 0.016
	R: GTGTTGCACATGGTAACG			
*recA*	F: GCAACGAAATGGTGGAACAG	147	[52]	1.98 ± 0.019
	R: AAGGCATCGGCAATCTCTAAG			
*ccfA*	F: CCGCAATTAAAGGCCTTGCAA	189	This study	1.98 ± 0.022
	R: TAACTCAGGCACGCGTGAAA			
*prgB*	F: AGCGGATGGAAAATTTTACTCACC	197	[53]	1.96 ± 0.086
	R: GCGCACTAGATACAGGCACATTA			
*prgQ*	F: ATGAAAACCACTCTAAAAAAAC	72	This study	1.99 ± 0.063
	R: TCAGATAAATATTAAGGTTATTGC			
*prgU*	F: AGAGAGGCAAAGGGGATGAAAG	127		1.97 ± 0.118
	R: TGTACCACATTTCCATTGCACG			
*prgX*	F: GCTGACTCTCGACCGATTTCT	200		1.99 ± 0.056
	R: CGCTTTGGCTCAATCCTTTG			

### Statistical analysis

All experiments, except scanning electron microscopy, were performed using three independently evolved lineages per condition. These lineages represent biological replicates generated through independent serial passaging. Data are presented as means ± standard error of the mean (SEM) of the independent lineages, representing the precision of the mean estimates for comparison against untreated controls. Statistical significance was assessed using unpaired *t*-test in GraphPad Prism v.8 (GraphPad Software, San Diego, CA, USA). Significance thresholds: ns (*p* ≥ 0.05); *p < 0.05 ; **p < 0.01; ***p < 0.001; ****p < 0.0001.

## Results

Donor and recipient strains were passaged 20x in the presence of ZnO, ZnONPs or in control conditions (see Fig. 1). Visible clumping and aggregation of the zinc-treated donor strains were first observed after approximately 15 passages. Although serial passaging enables investigation of long-term adaptation, this approach does not distinguish between physiological adaptation and selection of genetically altered subpopulations. Therefore, the observed phenotypic and transcriptional changes represent stable traits after prolonged zinc exposure but cannot be attributed exclusively to non-genetic regulation. All following experiments were performed with these passaged strains in the absence of ZnO or ZnONPs, except for the short-term zinc exposure conjugation assay. https://BioRender.com/f8goadc

### Long-term zinc exposure induces distinct phenotypic responses in *E. faecalis*

#### Bacterial growth

To evaluate the long-term impact of zinc stress on the fitness of *E. faecalis*, we initially analyzed growth kinetics and final cell densities. Optical density measurements over 24 h revealed reduced growth signal of zinc-treated *E. faecalis* OG1RF:pCF10 (Fig. 2A). Initial growth rates were comparable to the controls, but decline occurred earlier in zinc-treated cultures, leading to 52 ± 9% and 25 ± 13% lower OD₆₀₀ values for ZnO-treated and ZnONPs-treated cells, respectively. However, due to extensive cell aggregation observed in the zinc-treated cultures, OD₆₀₀ values likely do not fully reflect viable cell numbers. CFU-based growth analysis showed significantly lower recoverable CFU/mL in zinc-treated OG1RF:pCF10 cultures than in the control at 24 h (Fig. S1A).

#### Cell morphology

Macroscopic inspection revealed pronounced aggregation in zinc-treated conditions (Fig. S1B). ZnO-treated cultures formed larger aggregates with a tendency toward rapid sedimentation, whereas ZnONPs-treated cultures displayed smaller, more uniformly distributed clusters with slower sedimentation behavior. Zinc exposure had no significant effect on growth of *E. faecalis* OG1SSp (Fig. 2B). In line with this, scanning electron microscopy revealed increased aggregation and clumping in zinc-treated *E. faecalis* OG1RF:pCF10, accompanied by material consistent with extracellular structures surrounding the clusters (Fig. 2C), compared to the control. Quantitative analysis of cell width showed no statistically significant differences between control and zinc-exposed OG1RF:pCF10 cells (Table S1). Thus, prolonged zinc exposure in the donor strain was associated primarily with aggregation and extracellular matrix-like material rather than measurable changes in individual cell width. Recipient cells showed no apparent aggregation, clumping, or matrix-like structures (Fig. 2C). Consistent with the SEM observations, quantitative analysis of cell width showed no statistically significant differences between control and zinc-exposed OG1SSp cells (Table S1).

#### Metabolic activity within established biofilms

**Fig. 3:**
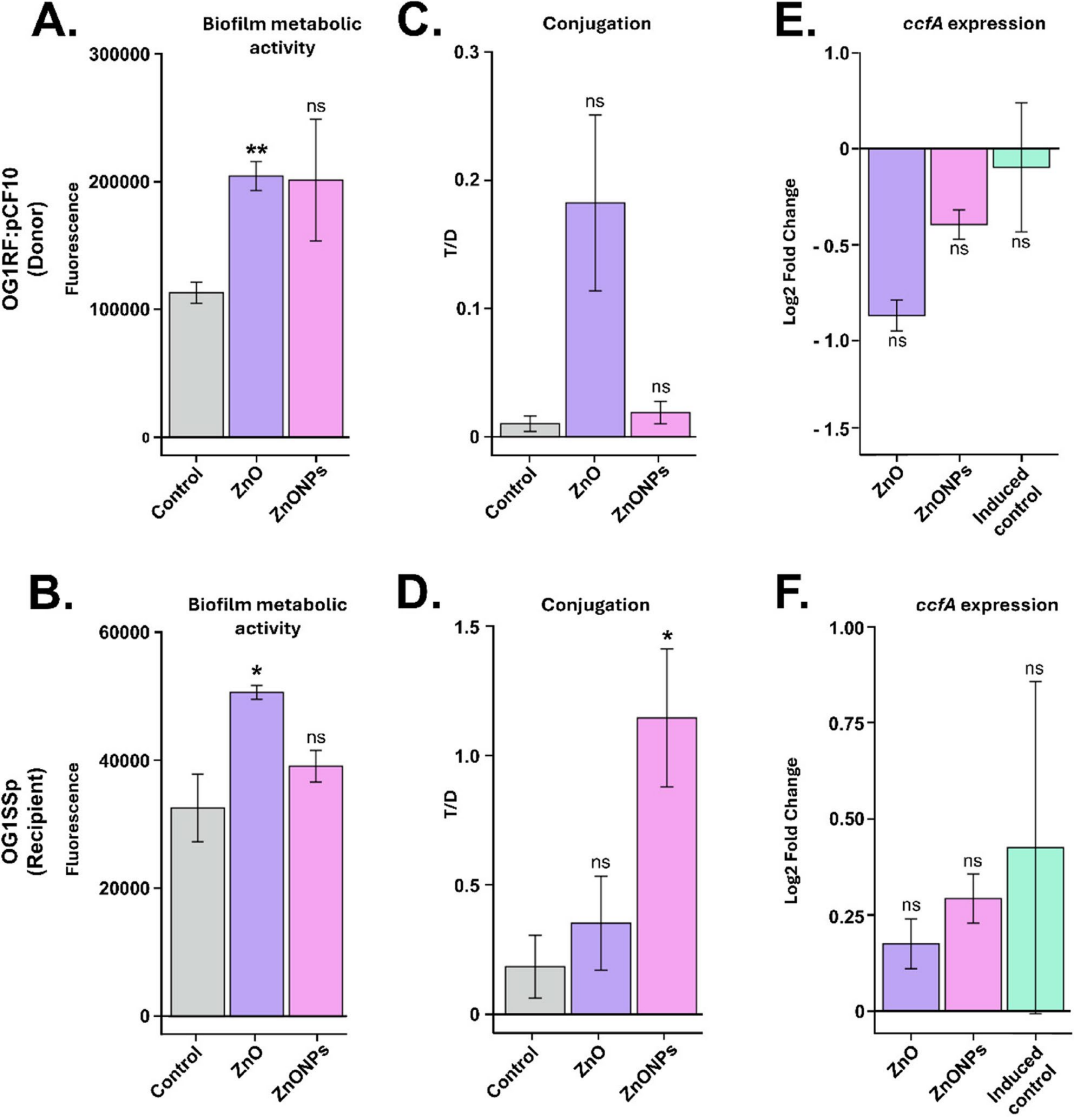
Phenotypic and transcriptional effects of long-term zinc exposure of *E. faecalis* OG1RFpCF10 and OG1SSp. **A**, **B** Metabolic activity of established biofilms in BHI supplemented with 1% glucose, with daily medium replacement. Biofilms were washed with PBS and assessed using Alamar Blue (excitation 560 nm, emission 590 nm). Each lineage was measured in eight technical replicates. Fluorescence-based readout reflects overall metabolic activity but does not distinguish between increased per-cell activity and increased cell numbers within the biofilm. **C**, **D** Conjugation efficiency expressed as transconjugants per donor (T/D) after 2.5 h mating at 37 °C following 2.5 h pre-incubation. (C) Treated donor (ZnO, ZnONPs, or control) mated with untreated recipient. **D** Treated recipient (ZnO, ZnONPs, or control) mated with untreated donor. **E**, **F** Expression of the chromosome encoded pheromone gene *ccfA*. Relative transcript levels were calculated as log₂ fold changes normalized to the reference genes *recA* and *gdhA*, using the respective untreated strain as the calibrator. Data are presented as mean ± SEM of three independently evolved lineages. Statistical significance was determined using unpaired t-test. *p < 0.05; **p < 0.01; ns, not significant

Given the observed cell aggregation and the presence of matrix-like structures, we subsequently investigated whether these changes translated into increased metabolic activity within established biofilms. Metabolic activity within established biofilms, assessed by Alamar Blue reduction, was significantly increased in both ZnO-treated *E. faecalis* strains (OG1RF:pCF10 and OG1SSp) compared to the untreated control. ZnONPs-treated cells of both strains showed a similar trend toward increased metabolic activity within biofilm-associated cells, but these differences were not statistically significant (Fig. 3A, B).

#### Gelatinase production

To determine whether the increased metabolic activity within established biofilms was associated with specific enzymatic virulence factors, gelatinase production was examined. Zinc (either form) exposure had no effect on gelatinase production by *E. faecalis* OG1RF:pCF10. Colonies produced clear halos on skim milk agar. Halo diameters were comparable across all treatments (Fig. S2). Gelatinase activity was not detected in any of the *E. faecalis* OG1SSp strains. The presence of the *gelE* gene was confirmed by gene-specific PCR in both strains.

#### Antibiotic sensitivity

To investigate whether zinc exposure induces cross-resistance to clinical antimicrobials, we performed disk diffusion testing. A panel of nine antibiotics was specifically selected according to EUCAST guidelines to represent the clinically relevant therapeutic options for *E. faecalis*. Testing with these agents, including β-lactams, macrolides/lincosamides, tetracyclines, sulfonamides, aminoglycosides, and glycopeptides revealed no differences between treated and untreated strains, with all values within the expected range of biological variability (Fig. S3).

### Long-term zinc exposure leads to altered conjugation and transcriptional responses in *E. faecalis*

The most prominent phenotypic adaptation, constitutive cell aggregation, is a hallmark of the pCF10-encoded conjugation system. We therefore examined whether zinc exposure altered HGT.

#### *E. faecalisOG1RF:pCF10*

Conjugation was evaluated by mating experiments in which ZnO-treated, ZnONPs-treated, and untreated *E. faecalis* OG1RF:pCF10 strains served as donors and untreated *E. faecalis* OG1SSp as the recipient. ZnO-treated donor cells resulted in an increase in transconjugant-per-donor (T/D) relative to the untreated control, whereas conjugation using ZnONPs-treated donors was comparable to that of the control (Fig. 3C). However, extensive cell aggregation in zinc-treated donor cultures resulted in increased variability, likely reflecting aggregation-related challenges in accurate colony enumeration.

**Fig. 4:**
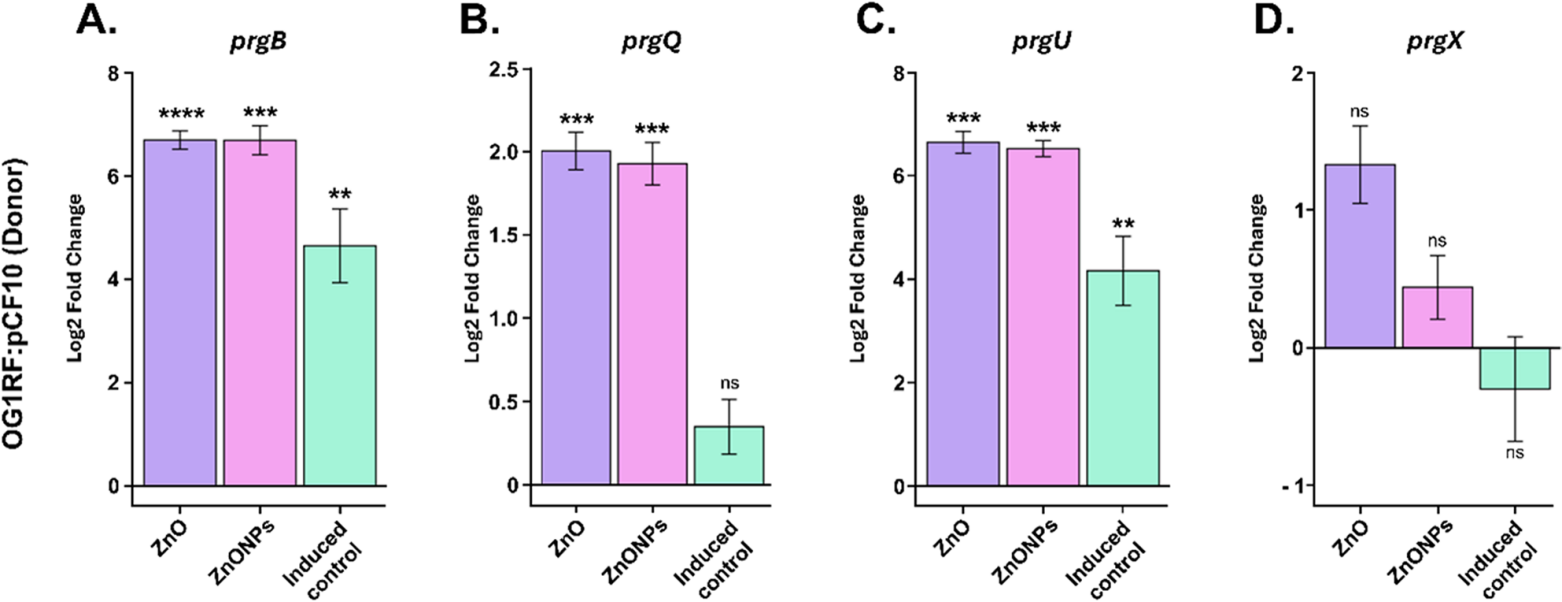
Expression of plasmid-encoded conjugation genes in *E. faecalis* OG1RF:pCF10 after long-term zinc exposure. Relative transcript levels were calculated as log₂ fold changes normalized to the reference genes *recA* and *gdhA* using the untreated donor as the calibrator. Panels show expression of *prgB* (**A**), *prgQ* (**B**), *prgU* (**C**), and *prgX* (**D**) in ZnO-treated, ZnONPs-treated, and induced control conditions (supernatant from untreated OG1SSp). RNA was isolated from strains after 20 serial passages and cultivated in BHI in the absence of ZnO or ZnONPs prior to analysis. Data are presented as mean ± SEM of three independently evolved lineages. Statistical significance was determined using an unpaired t-test. **p < 0.01; ***p < 0.001; ****p < 0.0001; ns, not significant

To investigate the molecular basis of these phenotypic observations, we quantified the transcription of specific genes involved in the pCF10 conjugation machinery, focusing on pheromone signaling and cell aggregation. The selected genes represent key regulatory and functional components of the pCF10 system: *prgQ* encodes the inhibitory pheromone iCF10 and reflects transcriptional activity within the *prgQ* regulatory region; *prgX* encodes the pheromone-responsive transcriptional regulator; *prgB* encodes the pCF10 aggregation substance involved in donor–recipient aggregation; and *prgU* is associated with protection against pheromone-induced overexpression toxicity. Analysis of transcript levels revealed upregulation of *prgB* and *prgU*, moderate induction of *prgQ*, but no significant changes in *prgX*, which is also coded on pCF10 but not as a part of the *prgQ* operon. To compare this response with the natural activation of the pCF10 system, an induced control was included, consisting of untreated donor cells stimulated by the cell-free supernatant of the recipient containing the cCF10 pheromone. The induced control showed a similar pattern at a lower level (Fig. 4). The expression of chromosomally encoded cCF10 pheromone gene *ccfA* was not significantly altered (Fig. 3E).

#### *E. faecalis *OG1SSp

To evaluate if long-term zinc exposure modified the efficiency with which the recipient acquires the pCF10 plasmid, conjugation assays were performed using zinc-treated OG1SSp cells. Conjugation efficiency increased relative to the untreated strain, with a statistically significant rise in the ZnONPs-treated group and a non-significant increase in the ZnO-treated cells (Fig. 3D).

To evaluate whether the increased recipient efficiency was associated with enhanced pheromone production, we quantified the transcription of the chromosomal *ccfA* gene encoding the cCF10 signaling pheromone. An induced control (untreated recipient exposed to donor supernatant) was included to assess whether recipient cells respond to donor-derived signals by altering their own pheromone production. Analysis of transcript levels revealed a slight, but non-significant increase in *ccfA* transcript levels in all groups compared to the untreated recipient (Fig. 3F).

### Short-term zinc exposure does not affect conjugation

To distinguish between stable adaptation following long-term exposure and immediate physiological responses to the metal, we conducted short-term exposure assays. Non-passaged *E. faecalis* OG1RF:pCF10 and OG1SSp were both exposed to ZnO, ZnONPs or DMSO (as control) for 2.5 h before mating and during the 2.5 h mating assay. In both treatment groups conjugation declined slightly, but not significantly, compared to the control (Fig. S4).

## Discussion

Zinc exposure represents a persistent environmental stressor with major consequences for bacterial physiology and evolution. Our previous studies in *Escherichia coli* demonstrated that long-term zinc stress induces phenotypic and molecular alterations, including enhanced virulence, multidrug resistance, and regulatory changes [45, 54]. Similar effects were reported in *Pseudomonas aeruginosa*, where zinc reshaped virulence and biofilm formation [55]. We show here that long-term zinc exposure in *E. faecalis* shifts phenotypes toward increased cell aggregation and conjugation, thereby linking zinc stress to changes in cellular aggregation, biofilm-associated metabolic activity, and horizontal gene transfer in Gram-positive bacteria.

Long-term zinc exposure did not lead to major changes in cell-shape, but induced aggregation and features consistent with extracellular matrix-like structures in *E. faecalis* OG1RF:pCF10, while the plasmid-free OG1SSp remained unaffected. This phenotype was observed specifically in zinc-treated, plasmid-carrying strains; however, whether it reflects plasmid-associated functions, altered surface properties, or genetic adaptation remains to be determined, although similar observations have been reported previously [56, 57]. The reduced OD₆₀₀ signal observed in zinc-treated *E. faecalis* OG1RF:pCF10 should therefore be interpreted with caution, as extensive aggregation and sedimentation can interfere with turbidity-based growth measurements. These findings are consistent with altered cellular homeostasis and an aggregation-prone phenotype [34, 36]. Importantly, a study by Honsa et al. [58] demonstrated that exposure to elevated zinc concentrations rapidly induced aggregation in *Streptococcus pneumoniae* accompanied by structural remodeling. This supports the concept that zinc promotes aggregation via envelope-mediated mechanisms [59]. Our CFU-based growth analysis also showed lower recoverable colony counts in zinc-treated cultures at 24 h. However, because these cultures displayed pronounced clumping, CFU enumeration should not be interpreted as a direct measure of the total number of viable individual cells. Aggregated cells may give rise to single colonies, thereby underestimating viable cell numbers. Therefore, the observed growth phenotype most likely reflects a combination of aggregation-related measurement effects and reduced CFU recovery, rather than providing definitive evidence for a major loss of viability.

Cells adapted to long-term zinc oxide exposure showed markedly increased metabolic activity within established biofilms, with the strongest effect observed in donor cells. Zinc oxide nanoparticles elicited the same overall trend but without statistical significance due to high variability. This is likely due to the inherent sensitivity of the biofilm assay where repeated medium replacement and washing steps over the 7-day period introduced high variability among eight replicates. This interpretation is consistent with the study of Deumić et al. [60]., who reported that sub-inhibitory concentrations of zinc salts enhanced biofilm formation in *E. faecalis*, possibly as part of an adaptive response to metal-induced stress. Supporting this notion, Abrantes et al. [56]. showed that *E. faecalis* expresses zinc-responsive proteins that counteract zinc overload. Given the strong association between biofilm and conjugation in *E. faecalis*, zinc-associated increases in metabolic activity within established biofilms may thus provide a microenvironment conducive to plasmid dissemination [61]. Importantly, the fluorescence-based assay used in this study determines overall metabolic activity within established biofilms and does not distinguish among metabolic activity per cell, a number of metabolically active biofilm-associated cells, or both [62]. Therefore, these data do not represent changes in total biofilm biomass.

No differences in gelatinase production were observed among treatments, which was only found to be active in donors, despite the presence of the *gelE* gene in both strains. This indicates that zinc stress did not activate proteolytic virulence pathways under the tested conditions [63].

Antibiotic susceptibility profiles remained unchanged across the tested zinc-treated and control strains of *E. faecalis*, indicating that ZnO and ZnONPs exposure did not directly induce phenotypic resistance to clinically relevant antibiotics [45]. This observation may reflect fundamental differences between Gram-positive and Gram-negative bacterial cells [64, 65]. In Gram-positive bacteria, metal exposure may preferentially promote horizontal gene transfer and virulence traits rather than *de novo* resistance acquisition [11, 12]. Together, these findings suggest that the primary risk associated with zinc in *E. faecalis* lies in the enhanced dissemination of existing resistance determinants through increased metabolic activity within established biofilms and conjugation.

**Fig. 5:**
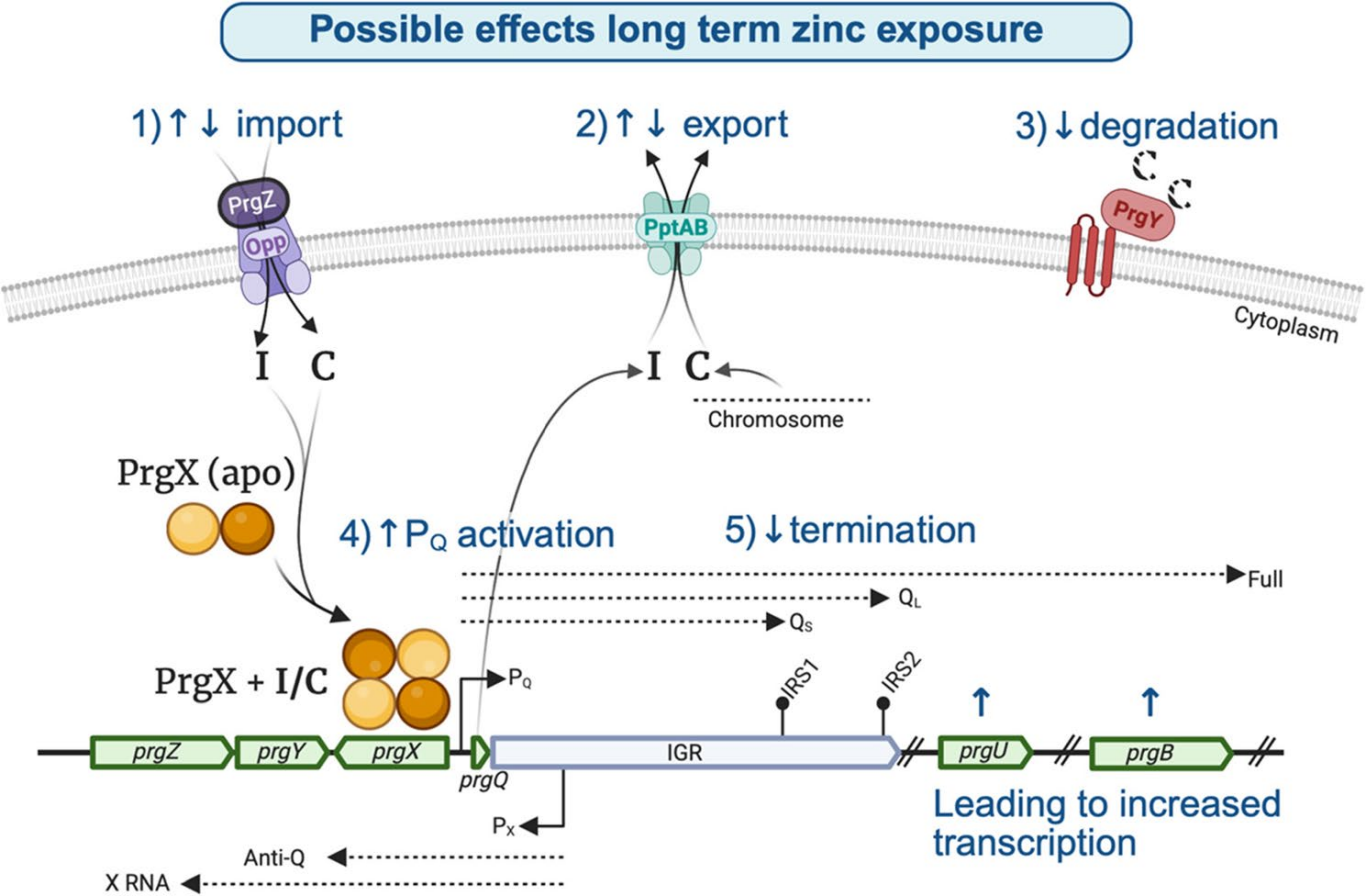
Schematic overview of the regulation of the *prgQ* operon in pCF10 and conceptual hypotheses for the effect of long-term zinc exposure. The pCF10 conjugation system is regulated by the balance between the inducing pheromone cCF10 (encoded on the chromosome by *ccfA*) and the inhibitory pheromone iCF10 (encoded by *prgQ*). Both pheromones are imported into donor cells via the PrgZ/Opp transport system and are subsequently sensed by the transcriptional regulator PrgX. cCF10 relieves repression of the *prgQ* promoter (PQ). This promotes transcriptional readthrough within the *prgQ* intergenic region (IGR). Transcription can terminate at the IRS1/2 sites (leading to Q_S_ or Q_L_ transcripts), or continue to produce Q_Full_, which contains all downstream conjugation genes, including prgB and prgU. Inhibitory pheromone iCF10, encoded by the *prgQ* gene, and the membrane protein PrgY, which cleaves donor-derived pheromone, prevent self-induction by donor cells. Long-term zinc exposure may be associated with increased transcription of the *prgQ* operon through multiple, non-exclusive hypothetical mechanisms. These include altered pheromone dynamics (1–3), such as changes in pheromone import (1), export (2), or degradation (3), thereby shifting the cCF10/iCF10 balance in favor of cCF10. Alternatively, zinc may increase transcription from the P_Q_ promoter (4) and/or reduce transcriptional termination within the regulatory IGR region (5), thereby facilitating readthrough and increased expression of conjugation-associated genes even under low or basal pheromone conditions. The model represents a conceptual framework based on the observed phenotypic and transcriptional data and on the established pCF10 regulatory system, but does not reflect experimentally confirmed mechanisms.Created in BioRender: https://BioRender.com/1wipkyp

Although ZnO-treated *E. faecalis* donor strain OG1RF:pCF10 showed an increase in conjugation frequency compared to the untreated control, this effect was not statistically significant. Nonetheless, the consistent upward trend combined with the higher transcription levels found for genes in the conjugative *prgQ* operon, is consistent with increased responsiveness of the conjugation system. In *E. faecalis* pCF10, the expression of the conjugative *prgQ* operon is tightly regulated and initiated by recipient-derived cCF10 pheromone [66, 67], encoded by ccfA on the *E. faecalis* genome. Self-induction is regulated amongst others by an inhibiting pheromone, iCF10, encoded by *prgQ*, the first gene in the *prgQ* operon on pCF10. Both pheromones are sensed by the transcriptional regulator PrgX [41, 44, 61]. Our transcriptional data of zinc-treated donors show activation of the *prgQ* operon with increased transcription of both *prgB and prgU*, while transcript levels for *prgX* (in an opposite reading frame) remain largely unchanged. The canonical pheromone regulation of the *prgQ* operon in pCF10 is illustrated in Fig. 5. The apparently lower increase of *prgQ* versus *prgB* transcript levels is likely due to the presence of a basal *prgQ* transcription from Q_S_ and Q_L_ transcripts that do not include *prgB* or *prgU* [14, 66]. Based on the observed transcriptional patterns, two non-exclusive hypotheses may explain the zinc-dependent increase of *prgB* and *prgU* expression. (i) Long-term zinc adaptation may have changed the cCF10/iCF10 pheromone balance and thereby enabled autocrine self-induction by endogenous cCF10 (“sensitization”; Fig. 5, processes 1–3), potentially via changed export by PptAB and/or import via PrgZ/Opp together with altered membrane properties and/or a reduction of cCF10 degradation by PrgY [17, 68, 69]. (ii) Long-term zinc exposure could relieve transcriptional attenuation by promoting antitermination within the *prgQ* region. This would lead to the production of extended *prgQ* operon transcripts even at basal levels of *prgQ* transcription, instead of premature termination within the regulatory region [19], consistent with the hypothesis that the operon can be activated independently of cCF10 (“bypass”; Fig. 5, processes 4–5). The data presented in this study are consistent with both hypotheses. The sensitization hypothesis (i) is biologically plausible based on the established roles of PrgB in aggregation and PrgU in protection from hyperinduction toxicity [14, 67, 70, 71], whereas the bypass hypothesis (ii) is compatible with zinc-exposed donors showing prgB/prgU amplitudes that exceed physiological pheromone stimulation. Consistently, environment-driven HGT has been demonstrated in communities: ZnONPs increase membrane permeability and oxidative stress [44], and the ionic liquid [BMIm][PF6] enhances RP4 transfer by suppressing the membrane barrier [72]. Overall, both routes converge on elevated levels of expression from the *prgQ* operon, leading to enhanced levels of PrgB, which is known to promote aggregation and biofilm-associated traits [67, 70]. These conceptual hypotheses generate testable predictions and provide a framework for future experiments.

Zinc-exposed recipient cells displayed significantly greater conjugation frequency, particularly under ZnONPs treatment. Since transcription of the pheromone gene *ccfA* in the recipient remained unchanged, this finding indicates that the enhanced plasmid uptake observed in zinc-adapted recipients is unlikely to be driven by transcriptional upregulation of *ccfA*. Instead, zinc may have altered cell-surface properties that facilitate mating, including envelope composition, electrostatic charge, and surface-associated molecules, thereby lowering barriers to mating pair formation and DNA transfer [44, 72–74]. The observed trend toward increased conjugation frequency, together with the strong transcriptional induction of *prgB* in zinc-treated donor cells, further supports the hypothesis that zinc stress enhances the responsiveness of the conjugation system. Wang et al. [44] demonstrated that ZnONPs facilitate horizontal transfer of resistance genes in bacterial communities by increasing membrane permeability and oxidative stress, thereby lowering the barriers for DNA exchange. A similar phenomenon was reported for other environmental pollutants, where ionic liquid [BMIm][PF6] significantly enhanced plasmid RP4 transfer in freshwater microcosms by suppressing the membrane barrier and increasing permeability [56, 72, 75]. This suggests that similar physicochemical mechanisms operate in *E. faecalis*, where ZnONPs create conditions favourable for recipient competence. However, no large changes of the cell surface or shape were observed by scanning electron microscopy, besides the increase in extracellular matrix material.

In contrast to long-term stress, short-term co-exposure of donor and recipient strains to zinc slightly but non-significantly reduced conjugation efficiency. This suggests that immediate toxic effects, such as membrane destabilization or oxidative imbalance, transiently outweigh any potential induction of the conjugation pathways [76]. Thus, zinc-driven enhancement of HGT appears to require long-term adaptation rather than acute exposure, as supported by the pronounced cell clumping phenotype observed after 15 passages under zinc stress.

Importantly, prolonged serial passaging under zinc stress may have introduced stable genetic changes. Although this approach allowed us to assess stable phenotypic and transcriptional outcomes after prolonged zinc exposure, it does not distinguish between physiological adaptation, stable regulatory adaptation, and selection of genetically altered subpopulations. Future studies should include whole-genome sequencing of control, ZnO-adapted, and ZnONP-adapted lineages to clarify the genetic basis of the zinc-adapted phenotype.

Our findings extend the established model of pheromone-induced conjugation and show that environmental stressors can also act as potent triggers of this process. Notably, the magnitude of *prgB* and *prgU* transcription under zinc exceeded pheromone-stimulated conditions and underscored the capacity of metal stress to interfere with endogenous signaling pathways. These results raise the possibility that pollutants such as zinc may reshape gene regulatory hierarchies and enhance horizontal gene transfer in the absence of canonical signals. 

## Conclusions

Our findings show that prolonged exposure to ZnO and ZnONPs is associated with reproducible phenotypic and transcriptional changes in Enterococcus faecalis, including aggregation, increased metabolic activity within established biofilms, and altered expression of genes within the *prgQ* operon. Increased conjugation efficiency in zinc-adapted recipient cells suggests that chronic zinc exposure may influence horizontal gene transfer dynamics. Future studies using whole-genome sequencing are needed to determine and differentiate the relative contribution of reversible physiological adaptation, stable regulatory adaptation, and genetic adaptation.

## Supplementary Information

Supplementary Material 1.

## Data Availability

The datasets generated and/or analysed during the current study are available from the corresponding author on request.
