# Supplementary material for: Prolonged zinc exposure modulates biofilm metabolic activity and conjugation in Enterococcus faecalis
Source: BMC Microbiol. 2026 Jun 4;26:704. doi: 10.1186/s12866-026-05250-x (PMC13449684; doi:10.1186/s12866-026-05250-x)
Supplement: Supplementary file 1 [file 12866_2026_5250_MOESM1_ESM.docx]

**Supplementary Information**

**Figure S1. Growth of *E. faecalis* OG1RF:pCF10 under zinc stress.**

(A) Growth dynamics based on CFU/mL over 24 h in untreated, ZnO-treated, and ZnONPs-treated cultures after 20 serial passages. Cultures were grown in Mueller–Hinton broth in the absence of ZnO or ZnONPs during measurement. Data are presented as mean ± SEM of three independently evolved lineages. Statistical significance at 24 h was determined using one-way ANOVA followed by Dunnett’s multiple comparisons test against the control. Significant differences compared with the control at 24 h are indicated. *p < 0.05; **p < 0.01.

(B) Macroscopic appearance of the cultures after 24 h, showing visible clump formation in zinc-treated samples. A black background was digitally added for contrast; the images were otherwise not modified.

**Table S1. Cell width of *E. faecalis* OG1RF:pCF10 and OG1SSp after long-term zinc exposure.**
Cell width was measured from SEM images shown in Fig. 2C after 20 serial passages under control, ZnO-treated, and ZnONPs-treated conditions. Twenty cells with clearly reliably identifiable lateral boundaries were measured per condition using Fiji/ImageJ after individual image calibration (n = 20). Data are presented as mean ± SEM. Statistical significance was determined using an unpaired t-test. No statistically significant differences were observed between ZnO- or ZnONPs-treated cells and untreated controls.

| **Strain** | **Treatment** | **Mean cell width ± SEM (µm)** | **p-value vs untreated control** |
| --- | --- | --- | --- |
| OG1RF:pCF10 | Control | 0.553 ± 0.008 | - |
| OG1RF:pCF10 | ZnO | 0.564 ± 0.005 | 0.261 |
| OG1RF:pCF10 | ZnONPs | 0.562 ± 0.005 | 0.341 |
| OG1SSp | Control | 0.576 ± 0.009 | - |
| OG1SSp | ZnO | 0.580 ± 0.004 | 0.693 |
| OG1SSp | ZnONPs | 0.572 ± 0.006 | 0.689 |

**Figure S2. Gelatinase activity of *E. faecalis* OG1RF:pCF10 after long-term zinc exposure.** Proteolytic activity was assessed by measuring the diameter of clearance zones on skim milk agar plates in untreated, ZnO-treated, and ZnONPs-treated cultures after 20 serial passages. Data are presented as mean ± SEM of three independently evolved lineages. Statistical significance was determined using an unpaired t-test. No statistically significant differences were observed between groups (ns, not significant).

**Figure S3. Antibiotic susceptibility of *E. faecalis* OG1RF:pCF10 and OG1SSp after long-term zinc exposure.** Disk diffusion assay results are shown as inhibition zone diameters (mm) for selected antibiotics after 20 serial passages. (A) OG1RF:pCF10. (B) OG1SSp. Data are presented as mean ± SEM of three independently evolved lineages. Statistical significance was determined using an unpaired t-test. No statistically significant differences were observed between treated and untreated controls (ns, not significant).

**Figure S4. Conjugation efficiency after short-term zinc exposure.** Both OG1RF:pCF10 (donor) and OG1SSp (recipient) cells were exposed to DMSO (control), ZnO or ZnONPs (2 mg/mL) immediately before mating and during the 2.5 h mating period. Conjugation efficiency is expressed as the ratio of transconjugants per donor (T/D). Data are presented as mean ± SEM of three independently evolved lineages. Statistical significance was determined using an unpaired t-test. No statistically significant differences were observed between treatments (ns, not significant).
